# Impact of COVID-19 on Patients Hospitalized with ST-Segment Elevation Myocardial Infarction in the United States during the Early Pandemic: An Analysis of Outcomes, Care Delivery, and Racial Disparities in Mortality

**DOI:** 10.3390/idr15010006

**Published:** 2023-01-06

**Authors:** Harris Majeed, Karthik Gangu, Rahul Shekhar, Shazib Sagheer, Ishan Garg, Hina Shuja, Aniesh Bobba, Prabal Chourasia, Sindhu Reddy Avula, Abu Baker Sheikh

**Affiliations:** 1 Department of Internal Medicine, University of New Mexico Health Sciences Center, Albuquerque, NM 87106, USA; 2 Department of Internal Medicine, University of Kansas Medical Center, Kansas City, KS 66103, USA; 3 Division of Cardiology, University of New Mexico Health Sciences Center, Albuquerque, NM 87106, USA; 4 Department of Medicine, Karachi Medical and Dental College, Karachi 74700, Pakistan; 5 Department of Medicine, John H. Stroger Jr. Hospital of Cook County, Chicago, IL 60612, USA; 6 Division of Hospital Medicine, Mary Washington Hospital, Fredericksburg, VA 22401, USA; 7 Department of Interventional Cardiology, Division of Cardiology, University of Kansas Health System St. Francis Campus, Kansas City, KS 66606, USA

**Keywords:** COVID-19, STEMI, PCI, CABG, coronary angiogram, mortality, national inpatient sample, United States

## Abstract

The COVID-19 pandemic has impacted healthcare delivery to patients with ST-segment elevation myocardial infarction (STEMI). The aim of our retrospective study was to determine the effect of COVID-19 on inpatient STEMI outcomes and to investigate changes in cardiac care delivery during 2020. We utilized the National Inpatient Sample database to examine inpatient mortality and cardiac procedures among STEMI patients with and without COVID-19. In our study, STEMI patients with COVID-19 had higher inpatient mortality (47.4% vs. 11.2%, aOR: 3.8, 95% CI: 3.2–4.6, *p* < 0.001), increased length of stay (9.0 days vs. 4.3 days, *p* < 0.001) and higher cost of hospitalization (USD 172,518 vs. USD 131,841, *p* = 0.004) when compared to STEMI patients without COVID-19. STEMI patients with COVID-19 also received significantly less invasive cardiac procedures (coronary angiograms: 30.4% vs. 50.8%, *p* < 0.001; PCI: 32.9% vs. 70.1%, *p* < 0.001; CABG: 0.9% vs. 4.1%, *p* < 0.001) and were more likely to receive systemic thrombolytic therapy (4.2% vs. 1.1%, *p* < 0.001) when compared to STEMI patients without COVID-19. Our findings are the result of complications of SARS-CoV2 infection as well as alterations in healthcare delivery due to the burden of the COVID-19 pandemic.

## 1. Introduction

Acute ST-segment elevation myocardial infarction (STEMI) is a cardiac emergency that requires timely reperfusion [1]. The coronavirus disease-2019 (COVID-19) pandemic, caused by severe acute respiratory syndrome-coronavirus-2 (SARS-CoV2), has adversely impacted the delivery of healthcare to patients with STEMI [1,2]. Several care-delivery process changes were observed across the world during this period, including alternative cardiac reperfusion protocols, supply chain shortages, redirection of resources, and delays in patient presentation as well as intervention [3]. This observation is complicated by the fact that patients with COVID-19 have more cardiovascular complications as a sequalae of primary SARS-CoV2 infection, including increased risk of thrombosis, myocarditis, and pericarditis, all of which can also present with electrocardiographic ST segment elevations and increased serum troponins [4,5]. Based on this information, we postulate that STEMI outcomes for hospitalized patients were likely poorer during the early phases of the COVID-19 pandemic.

There is limited data suggesting that outcomes for STEMI patients during the early pandemic were inferior to pre-pandemic standards of care; one large database study (n = 82,640) by Saad et al., 2021, showed that STEMI patients with COVID-19 had significantly higher rates of in-hospital mortality (78.5% vs. 46.1%, OR: 4.11, 95% CI: 2.97–5.69, *p* < 0.001) when compared to STEMI patients without COVID-19 [6]. The aim of our study is twofold: (1) to determine the consequence of COVID-19 infection on inpatient STEMI mortality and other clinical outcomes among hospitalized patients in the United States during 2020 in the early stages of the pandemic, and (2) to investigate whether changes to the delivery of cardiac care during 2020, especially invasive cardiac procedures, contributed to the observed outcomes by comparing utilization to hospitalized STEMI patients without COVID-19 during the same time period using a large, representative sample from the United States National Inpatient Sample (NIS) database.

## 2. Materials and Methods

This retrospective study utilized the Agency for Healthcare Research and Quality (AHRQ) 2020 NIS database, which is based on hospitalizations from 1 January 2020 to 31 December 2020 [7]. NIS is the largest publicly available all-payer inpatient care database in the United States, containing data on more than seven million hospital stays [7]. The NIS database contains demographic information, medical comorbidities, in-hospital outcomes, procedures, and other discharge-related information for hospitalizations across the United States. NIS data for the year 2020 was released in September of 2022. In this study, all patients who were 18 years of age and older and were admitted to the hospital with STEMI were included; they were further stratified by COVID-19 status. International classification of diseases 10th—clinical modification (ICD-10-CM) codes were used to retrieve patient samples with comorbid conditions and ICD-10 procedure codes were used to identify inpatient procedures. A detailed code summary is provided in Supplementary Table S1. Patients who were under the age of 18 years or were transferred out of the index hospital were excluded from our study.

### 2.1. Baseline Charactertistics

Baseline characteristics related to patient demographics and comorbidities as well as hospital characteristics for all included patients were extracted from the NIS database, as detailed below:

(a) Patient characteristics: sex, age, race, insurance status, median income based on zip code, medical comorbidities, and Elixhauser comorbidity score.
(b) Hospital characteristics: geographic division, teaching status, and size.

### 2.2. Primary and Secondary Study Outcomes

Study outcomes were also obtained from the NIS database. The primary outcome of our study was in-hospital mortality. Secondary outcomes were as follows:

(a) Length of stay.
(b) Cost of hospitalization.
(c) Utilization of intubation and mechanical ventilation.
(d) Utilization of vasopressors.
(e) Incidence of cardiogenic shock.
(f) Disposition at discharge.
(g) Utilization of coronary angiograms.
(h) Utilization of percutaneous coronary intervention (PCI), coronary artery bypass grafting (CABG), or systemic thrombolytic therapy for coronary revascularization.
(i) Utilization of percutaneous left ventricular assist device (pLVAD) or intra-aortic balloon pump (IABP).
(j) Utilization of temporary transvenous pacing or permanent pacemaker.

### 2.3. In-Hospital Cardiovascular Complications

In addition to our primary and secondary outcomes, we examined cardiovascular complications associated with STEMI. We used ICD-10 codes (outlined in Supplementary Table S1) to identify the following complications:

(a) Arrhythmias: atrial fibrillation, atrial flutter, ventricular tachycardia, ventricular fibrillation, sinus bradycardia, first degree atrioventricular nodal block, second degree atrioventricular nodal block, and third degree atrioventricular nodal block.
(b) Pericarditis.

### 2.4. Statistical Methods and Covariate Selection

STATA 17 (StataCorp LLC, College Station, TX, USA) was utilized for statistical analysis. The unweighted sample was 6.34 million observations, and the weighted sample was 31.71 million discharges for the year 2020. Patients who were hospitalized with STEMI were retrieved from the NIS database using ICD-10-CM codes and was divided into two cohorts based on COVID-19 status. The described patient characteristics, hospital characteristics, primary and secondary outcomes, and in-hospital cardiovascular complications were extracted for each patient.

Chi-square analysis was used to compare categorical variables and linear regression was used to compare continuous variables among both study cohorts (STEMI with COVID-19 vs. STEMI without COVID-19). For our primary outcome, in-hospital mortality, we utilized a multivariate logistic regression model to adjust for potential confounders; a two-tailed *p*-value of less than 0.05 was considered significant. This multivariate logistic regression model was built using covariates with unadjusted *p*-values of less than 0.2 on initial univariate regression. We arbitrarily chose a *p*-value of less than 0.2 for covariate inclusion to broaden the number of variables accounted for by our regression model and to decrease the probability of missing potential confounders that may impact the standard error for our exposure variable (COVID-19 infection). Ultimately, our multivariate analysis adjusted for sex, age, race, median household income, insurance status, hospital geographic division, Elixhauser comorbidity score, need for mechanical ventilation, and numerous other in-hospital complications as denoted (*) in Table 1 and Table 4. Similarly, for our other secondary continuous outcomes, length of stay and cost of hospitalization, multivariate linear regression was used; a two-tailed *p*-value of 0.05 was considered significant. Finally, multivariate logistic regression was also utilized for adjusted analysis of secondary outcomes related to illness severity (incidence of cardiogenic shock, utilization of vasopressors and mechanical ventilation) and secondary therapeutic procedural outcomes.

## 3. Results

### 3.1. Baseline Characteristics

We identified 172,185 patients with STEMI between 1 January 2020 and 31 December 2020. Of these, 5786 patients had a diagnosis of COVID-19 (3.4%). STEMI patients with COVID-19 were significantly older (46% vs. 35%, *p* < 0.001, of patients were above 70 years of age), had a greater proportion of Hispanics (23.3% vs. 8.9%, *p* < 0.001) and African Americans (16.0% vs. 10.3%, *p* < 0.001), and were more likely to have household incomes below $50,000 (37.0% vs. 28.4%, *p* < 0.001) when compared to STEMI patients without COVID-19. While there was variability in the geographic distribution of patients in both cohorts, there were no significant differences in hospital teaching status, in that most STEMI patients regardless of COVID-19 status were seen at urban teaching hospitals. Additionally, STEMI patients with COVID-19 had a higher proportion of Medicare beneficiaries (56.5% vs. 50.1%, *p* < 0.001) and a lower proportion of private health insurance beneficiaries (26.3% vs. 32.0%, *p* < 0.001) when compared to STEMI patients without COVID-19.

STEMI patients with COVID-19 had a lower prevalence of cardiac comorbidities, including coronary artery disease (55.2% vs. 78.9%, *p* < 0.001), congestive heart failure (39.8% vs. 42.7%, *p* = 0.04), uncomplicated hypertension (35.0% vs. 41.8%, *p* < 0.001), and smoking (48.6% vs. 28.1%, *p* < 0.001) when compared to STEMI patients without COVID-19. Conversely, this group had a higher proportion of complicated hypertension (41% vs. 34.6%, *p* < 0.001), complicated diabetes mellitus (33.1% vs. 20.8%, *p* < 0.001), and chronic kidney disease (26.4% vs. 17.3%, *p* < 0.001)**.** There were no significant differences in the rates of chronic pulmonary disease or obesity between the two groups. Table 1 outlines the baseline characteristics of both study cohorts.

### 3.2. In-Hospital Mortality, Subgroup Analysis, and Secondary Non-Cardiac Procedural Outcomes

After multivariate adjustment, we found that the in-hospital mortality of STEMI patients with COVID-19 was significantly higher than STEMI patients without COVID-19 (47.4% vs. 11.2%, aOR: 3.8, 95% CI: 3.2–4.6, *p* < 0.001).Additionally, we noted that STEMI patients with COVID-19 required mechanical ventilation (43.7% vs. 15.2%, aOR: 3.8, 95% CI: 3.3–4.5, *p* < 0.001) and vasopressors (9.7% vs. 3.9%, aOR: 1.6, 95% CI: 1.3–2.1, *p* < 0.001) at higher rates despite a lower adjusted incidence of cardiogenic shock (14.2% vs. 13.4%, aOR: 0.7, 95% CI: 0.6–0.9, *p* = 0.003) when compared to STEMI patients without COVID-19. STEMI patients with COVID-19 also had an increased length of stay (9.0 days vs. 4.3 days, adjusted length of stay: 3.1 days greater, *p* < 0.001) and a higher cost of hospitalization (USD 172,518 vs. USD 131,841, inflation-adjusted cost of hospitalization: USD 29,687 higher, *p* = 0.004) Of those patients who survived, fewer STEMI patients with COVID-19 were able to return home (61.8% vs. 77.0%, *p* < 0.001) and more required skilled nursing or long-term acute care (22.1% vs. 9.7%, *p* < 0.001) when compared to STEMI patients without COVID-19. Table 2 outlines the primary and secondary non-cardiac procedural outcomes of both study cohorts.

We also performed subgroup mortality analysis and found that among STEMI patients with COVID-19, Hispanics (23.3% vs. 9.2%, *p* < 0.001), African Americans (16.6% vs. 12.1%, *p* = 0.005), and Native Americans (1.0% vs. 0.4%, *p* = 0.02) had increased in-hospital mortality when compared to Hispanic, African American, and Native American STEMI patients without COVID-19, respectively. Conversely, a significantly lower proportion of Caucasians in the STEMI with COVID-19 cohort had in-hospital mortality when compared to Caucasians in the STEMI without COVID-19 cohort (48.7% vs. 71.2%, *p* < 0.001). Table 3 outlines mortality subgroup analysis.

### 3.3. Inpatient Cardiac Procedures, Interventions, and Cardiovascular Complications

Notably, STEMI patients with COVID-19 were more likely to receive systemic thrombolytic therapy when compared to STEMI patients without COVID-19 (4.2% vs. 1.1%, aOR: 3.2, 95% CI: 2.2–4.6, *p* < 0.001). Apart from this, STEMI patients with COVID-19 were significantly less likely to receive all types of invasive cardiac interventions including coronary angiography (30.4% vs. 50.8%, *p* < 0.001), percutaneous coronary interventions (32.9% vs. 70.1%, aOR: 0.3, 95% CI: 0.3–0.3, *p* < 0.001), coronary artery bypass grafting (0.9% vs. 4.1%, aOR: 0.3, 95% CI: 0.1–0.5, *p* < 0.001), intra-aortic balloon pumps (4.1% vs. 6.5%, aOR: 0.6, 95% CI: 0.4–0.9, *p* = 0.002), percutaneous left ventricular assist devices (1.2% vs. 2.9%, aOR: 0.4, 95% CI: 0.2–0.7, *p* < 0.001), and transvenous pacing (1.2% vs. 2.6%, *p* = 0.005) when compared to STEMI patients without COVID-19. Table 4 outlines the distribution of cardiac procedures among both study cohorts. Supplementary Table S2 shows the utilization of therapeutic cardiac procedures after multivariate adjustment for both cohorts. STEMI patients with COVID-19 also had significantly higher rates of atrial flutter (3.1% vs. 2.1%, *p* = 0.02), atrial fibrillation (17.3% vs. 13.6%, *p* < 0.001), and ventricular fibrillation (5.8% vs. 8.1%, *p* = 0.004). Table 4 also outlines the distribution of cardiac complications among both study cohorts.

## 4. Discussion

In our US-based study, hospitalized STEMI patients with concomitant COVID-19 infection had a significantly higher rate of inpatient mortality when compared to hospitalized STEMI patients without COVID-19 infection during the early pandemic and had an accompanying increase in length of stay and cost of hospitalization. STEMI patients with COVID-19 also received significantly less invasive cardiac procedures (both diagnostic and therapeutic) and were instead more likely to receive systemic thrombolytic therapy.

Moreover, there are clinical markers to indicate that STEMI patients with COVID-19 were more critically ill, including higher levels of vasopressor and mechanical ventilatory dependence despite similar levels of cardiogenic shock. The higher mortality observed among STEMI patients with COVID-19 was also despite lower levels of pre-existing cardiac comorbidities prior to the presenting STEMI. Thus, we believe that the etiology of the observed marked increase in mortality is likely multi-factorial—related both to the infectious and cardiovascular complications of COVID-19 infection as well as system-wide alterations in delivery of care resulting in significantly fewer cardiac procedures. While our findings are consistent with prior literature, to our knowledge, this study has the largest sample size and shows a dramatic difference in STEMI mortality and cardiac procedural utilization between patients with COVID-19 and patients without COVID-19.

The mortality of COVID-19 due to acute infectious complications such as respiratory failure and septic shock is well-characterized. However, there is a growing body of literature establishing the role of acute SARS-CoV2 infection in the development of acute cardiovascular complications, which include various forms of myocardial injury (myocarditis, stress cardiomyopathy, and myocardial infarction), congestive heart failure, and cardiac arrhythmia [8,9]. The proposed mechanisms of COVID-19-mediated cardiovascular pathophysiology include [10,11,12]:

- Myocardial injury from hemodynamic instability or hypoxemia;
- Inflammatory myocarditis;
- Stress cardiomyopathy;
- Microvascular dysfunction;
- Thrombosis with coronary artery plaque destabilization due to inflammatory hypercoagulability.

Observational studies suggest that COVID-19 increases the risk of acute myocardial infection (AMI); one analysis found that the risk of AMI in patients with COVID-19 is significantly elevated (0.03% vs. 0.01%, aOR: 1.22, 95% CI: 1.08–1.38) [13]. There is also evidence of subacute cardiac complications after the first 30 days of SARS-CoV2 infection, including increased incidence of dysrhythmias, ischemic and non-ischemic cardiomyopathies with congestive heart failure, pericarditis, myocarditis, and thromboembolic disease [14,15]. Furthermore, there is a bidirectional relationship between COVID-19 infection and coronary artery disease (CAD) that is worth acknowledging; the risk of severe COVID infection increases considerably in those with pre-existing coronary artery disease [16]. According to a report released by the Center for Disease Control and Prevention (CDC) based on a Chinese patient population in early 2020, 4.2% of all COVID-19 patients had a known diagnosis of coronary heart disease, which increased to 22.7% among fatal cases of COVID-19 [17]. With this mind, it is probable that a portion of COVID-19 patients had undiagnosed CAD with an index STEMI presentation precipitated by SARS-CoV2 infection. Finally, the mortality observed during 2020 is likely affected by the lack of COVID-19 therapeutics; glucocorticoids were underutilized and remdesivir, tocilizumab, and mRNA vaccinations only became available in the later course of the pandemic and significantly reduced mortality [18].

With respect to the lower utilization of invasive cardiac procedures among STEMI patients with COVID-19 and its relation to the observed increase in mortality for this cohort, there are several possible explanations. Firstly, a major contributing factor is the strain of the COVID-19 pandemic on systems of healthcare delivery [6]. Throughout various stages of the pandemic there have been critical supply shortages; as it pertains to STEMI care, shortages in heparin, iodinated contrast, anesthetic agents for moderate sedation (midazolam and fentanyl citrate) troponin assays, SARS-CoV2 rapid PCR testing, N95 respirators, and personal protective equipment are thought to be particularly impactful for cardiac catheterization and other bedside cardiac procedures [19,20,21]. Similarly, redirection of inpatient resources, catheterization lab unavailability, and ancillary staffing deficiencies are likely notable contributors [22]. A second explanation of decreased cardiac procedural utilization may be that referring clinicians and cardiologists felt that STEMI patients with COVID-19 were too critically ill and less likely to benefit from invasive cardiac interventions such as coronary angiography and PCI due to poorer prognosis from acute infection. Our data lend support to this notion, as STEMI patients with COVID-19 had significantly increased mechanical ventilatory and vasopressor dependence. Correspondingly, patients with critical illness due to COVID-19, commonly characterized by hemodynamic instability, circulatory shock, and respiratory failure with mechanical ventilation, are suboptimal surgical candidates for CABG. Another interesting theory revolves around the underlying pathophysiology of COVID-19 associated STEMI. Studies have noted that COVID-19 patients can present with STEMI, but without obstructive CAD, and may account for up to 17% of STEMI cases [23]. These non-obstructive myocardial infarctions are potentially caused by direct vascular injury, hypoxia-induced injury, or viral invasion. Patients with non-obstructive disease may not benefit from revascularization via PCI or CABG. While diagnostic error is worth considering, the higher rate of systemic thrombolytic therapy use among STEMI patients with COVID-19 suggests that obstructive myocardial infarction remained on the differential for these patients.

We also found both economic and racial disparities in mortality. Hispanics, African Americans, and those with lower household income had increased mortality when hospitalized with STEMI and COVID-19, which is consistent with prior literature [24]. As expected, mortality of STEMI patients increased with age regardless of COVID-19 status. STEMI mortality was also significantly higher at urban teaching hospitals regardless of COVID-19 status and is likely an indicator of the acuity and comorbidity burden of this patient population [25]. We observed a higher mortality in Caucasian STEMI patients without COVID-19 in comparison to Caucasian STEMI patients with COVID-19; this finding is a departure from previous literature and presents an opportunity for further analysis. Currently, there is no evidence to suggest that COVID-19 infection is protective during acute STEMI in Caucasian patients, and our finding is likely more reflective of the poor outcomes in Caucasian STEMI patients overall.

Our study has several limitations. The first is possible detection bias, particularly as it relates to COVID-19 infection. The ICD-10 diagnosis code for COVID-19 was not developed until late 2020, which allows for the possibility that certain COVID-19 diagnoses were not captured in our dataset. Consequently, a subset of patients with STEMI may have had COVID-19, but were included in the non-COVID-19 cohort due to under diagnosis. Likewise, COVID-19 testing remained in short supply and the NIS database does not provide lab test results to confirm COVID-19 status. This principle applies to a lesser extent to inaccurately documented or undocumented comorbid diagnoses. The second limitation is that selection bias and confounding remain a possibility in this retrospective study. Our statistical analysis accounted for numerous demographic and clinical factors via multivariable logistic regression—however unaccounted variables may underlie our findings. This dataset did not include certain clinical, biochemical, or radiographic markers of COVID-19 severity which may conceivably have contributed more heavily to the observed mortality rather than the STEMI diagnosis.

Based on this study, we recommend further examination of the cardiac care delivery process during the early pandemic. Future investigations should evaluate process measures that explore time-to-intervention, procedural complications, and barriers to definitive therapies such as PCI and CABG.

We conclude that hospitalized STEMI patients with concomitant COVID-19 infection were nearly four times as likely to die in the hospital in comparison to STEMI patients without COVID-19 during 2020, in the early stages of the COVID-19 pandemic. This finding is likely driven by both clinical complications of COVID-19 infection as well as system-wide alterations in delivery of cardiac care due to the healthcare burden of the COVID-19 pandemic. Thus, in addition to focusing on risk mitigation strategies for preventing infection during future pandemics, it is equally important to develop strategies for maintaining other critical clinical processes to allow for the delivery of healthcare to patients with life-threatening conditions such as STEMI in order to reduce unnecessary morbidity and mortality.

## Figures and Tables

**Table 1:** Baseline characteristics of STEMI patients, based on COVID-19 status.

Characteristics	STEMI with COVID-19	STEMI without COVID-19	*p*-Value
n = 172,185	n = 5786 (3.4%)	n = 166,399 (96.6%)	
Female Sex *	33.8%	32.6%	0.39
Age Groups *			
≥18–29	0.5%	0.4%	
30–49	9.2%	12.5%	
50–69	44.3%	51.4%	
≥70	46.1%	35.7%	
Race *			<0.001
Caucasian	50.7%	73.5%	
African American	16.0%	10.3%	
Hispanic	23.3%	8.9%	
Asian or Pacific Islander	4.1%	3.0%	
Native American	1.0%	0.5%	
Other	4.9%	3.8%	
Median Household Income *			<0.001
<USD 50,000	37.0%	28.4%	
USD 50,000–64,999.99	26.0%	27.8%	
USD 65,000–86,000	21.3%	23.6%	
>USD 86,000	15.8%	20.2%	
Insurance Status *			<0.001
Medicare	56.5%	50.1%	
Medicaid	12.0%	11.2%	
Private	26.4%	32.0%	
Self-pay	5.2%	6.7%	
Hospital Geographic Division *			<0.001
New England	3.6%	4.3%	
Middle Atlantic	14.6%	11.9%	
East North Central	14.3%	15.3%	
West North Central	6.3%	6.3%	
South Atlantic	16.3%	22.2%	
East South Central	5.6%	7.8%	
West South Central	17.8%	12.1%	
Mountain	7.7%	7.5%	
Pacific	13.8%	12.5%	
Hospital Size			<0.001
Small	24.5%	19.0%	
Medium	29.0%	30.8%	
Large	46.6%	50.2%	
Hospital Teaching Status			0.09
Rural	9.4%	7.6%	
Urban non-teaching	18.7%	19.4%	
Urban teaching	71.9%	73.0%	
Medical Comorbidities			
Coronary artery disease	55.2%	78.9%	<0.001
Congestive heart failure	39.8%	42.7%	0.04
Hypertension, uncomplicated	35.0%	41.8%	<0.001
Hypertension, complicated	41.0%	34.6%	<0.001
Diabetes mellitus, uncomplicated	16.9%	14.8%	0.06
Diabetes mellitus, complicated	33.1%	20.8%	<0.001
Chronic kidney disease	26.4%	17.3%	<0.001
Chronic pulmonary disease	17.9%	17.2%	0.56
Obesity	18.8%	19.8%	0.38
Smoking	28.1%	48.6%	<0.001

**Table 2:** STEMI Primary and Secondary Non-Cardiac Procedural Outcomes Based on COVID-19 Status.

Outcomes	STEMI with COVID-19	STEMI without COVID-19	*p*-Value
In-hospital mortality (n = 21,385)	47.4%	11.2%	<0.001
	Adjusted odds ratio: 3.8 (95% CI: 3.2–4.6)		
Mean cost of hospitalization (USD)	USD 172,518	USD 131,841	0.004
	Adjusted total cost: USD 29,687 higher		
Mean length of stay (days)	9.0 days	4.3 days	<0.001
	Adjusted length of stay: 3.1 days more		
Cardiogenic shock	14.2%	13.4%	0.003
	Adjusted odds ratio: 0.7 (95% CI: 0.6–0.9)		
Vasopressor use	9.7%	3.9%	<0.001
	Adjusted odds ratio: 1.6 (95% CI: 1.3–2.1)		
Mechanical ventilation	43.7%	15.2%	<0.001
	Adjusted odds ratio: 3.8 (95% CI: 3.3–4.5)		
Disposition			<0.001
Home without health care	61.8%	77.0%	
Skilled nursing facility or long-term acute care	22.1%	9.7%	
Home with health care	13.4%	11.9%	
Against medical advice	2.8%	1.5%	

**Table 3:** STEMI In-Hospital Mortality Sub-Group Analysis Based on COVID-19 Status.

Variable	STEMI with COVID-19	STEMI without COVID-19	*p*-Value
Inpatient mortality (n = 21,385)	n = 2740	n = 18,645	
Sex			0.007
Male	64.2%	58.0%	
Female	35.77%	42.0%	
Age Groups			
≥18–29	0.18%	0.5%	0.32
30–49	5.11%	5.9%	0.45
50–69	38.87%	37.6%	0.57
≥70	55.84%	56.0%	0.93
Race			
Caucasian	48.47%	71.2%	<0.001
African American	16.6%	12.1%	0.005
Hispanics	23.33%	9.2%	<0.001
Asian or Pacific Islander	4.05%	3.5%	0.10
Native American	0.99%	0.4%	0.02
Other	4.86%	3.7%	0.24
Hospital Teaching Status			
Rural	8.58%	7.4%	0.33
Urban non-teaching	20.26%	19.5%	0.66
Urban teaching	71.17%	73.2%	0.32

**Table 4:** Inpatient Procedures and Cardiovascular Complications Among Hospitalized STEMI Patients, Stratified by COVID-19 Status.

Outcome	STEMI with COVID-19	STEMI without COVID-19	*p*-Value
Inpatient Procedures			
Coronary angiography	34.1%	74.2%	<0.001
Percutaneous coronary intervention *	32.9%	70.1%	<0.001
Coronary artery bypass grafting *	1.0%	4.1%	<0.001
Systemic thrombolytic therapy *	4.2%	1.1%	<0.001
Intra-aortic balloon pump *	4.2%	6.5%	0.002
Percutaneous left ventricular assist device *	1.2%	2.9%	<0.001
Transvenous pacing *	1.3%	2.6%	0.005
Permanent pacemaker placement *	0.2%	0.6%	0.090
Cardiovascular Complications			
Pericarditis *	0.6%	0.5%	0.570
Atrial fibrillation *	17.3%	13.6%	<0.001
Atrial flutter *	3.1%	2.1%	0.020
Ventricular fibrillation *	5.8%	8.1%	0.004
Ventricular tachycardia *	12.4%	13.5%	0.290
Sinus bradycardia *	4.5%	5.9%	0.040
First-degree atrioventricular block	1.5%	1.1%	0.180
Second-degree atrioventricular block	0.8%	0.8%	0.900
Third-degree atrioventricular block *	2.1%	3.0%	0.070

* Incorporated into multivariate analysis.

## Data Availability

Not applicable.

## Supplementary Materials

The following supporting information can be downloaded at: https://www.mdpi.com/article/10.3390/idr15010006/s1, Supplemental Table S1: ICD-10 Codes For Diagnosis and Procedures; Supplemental Table S2: Utilization of Therapeutic Cardiac Procedures After Multivariate Adjustment Among Hospitalized STEMI Patients, Stratified by COVID-19 Status.
