# Supplementary material for: Impact of COVID-19 on Patients Hospitalized with ST-Segment Elevation Myocardial Infarction in the United States during the Early Pandemic: An Analysis of Outcomes, Care Delivery, and Racial Disparities in Mortality
Source: Infect Dis Rep. 2023 Jan 6;15(1):55–65. doi: 10.3390/idr15010006 (PMC9844427; doi:10.3390/idr15010006)
Supplement: Supplementary file 1 [file idr-15-00006-s001.zip › idr-2027556-supplementary.pdf]

Supplemental Table S1: ICD-10 codes

Variable	ICD-10 CM code
STEMI	I21.0X, I21.1X, I21.2X, I21.3X, I21.9X
Covid	U071, U00, U49, U50, U85, J1282
Cardiogenic shock	R570
Pericarditis	I24.1
A.fib	I48.0, I48.1, I48.91
A.flutter	I48.3, I48.4, I48.92
V.fib	I49.01, I49.02
Vt	I47.2
Sinus brady	R00.1
First degree av block	I44.0
Second degree av block	I44.1
Third degree av block	I44.2
Smoking	F17.XX, Z87.891
CAD	I25.10, I25.11, I25.118, I25.119, I252, I253, I25.4XX, I25.5, I25.6, I25.8XX, I25.7XX, I25.9XX
CHF, HTN, DM, Renal failure, Chronic pulmonary disease, Obesity	Elixhauser comorbidities were used
**Variable**	**ICD-10 procedure code**
Intubation	5A1945Z, 5A1955Z, 5A1935Z, 5A09357, 5A09457, 5A09557
tPA use	3E03317, 3E04317, 3E05317, 3E06317, 3E08317
Vasopressor use	3E030XZ, 3E033XZ, 3E040XZ, 3E043XZ, 3E050XZ, 3E053XZ, 3E060XZ, 3E063XZ
CABG	0210XX, 0211XX, 0212XX, 0213XX
PCI	0203XX, 02704XX, 02713XX, 02714XX, 02723XX, 02724XX, 02733XX, 02734XX, 02C03XX, 02C04XX, 02C13XX, 02C14XX, 02C23XX, 02C24XX, 02C33XX, 02C34XX
Coronary angiography	B217XX, B218XX, B21FXX, B210XX, B211XX, B212XX, B213XX
IABP	5A02210, 5A02110
IMPELLA	5A02116, 5A0211D, 5A02216, 5A0221D
Transvenous pacing	5A1223Z, 5A1213Z
Pacemaker insertion	0J5604Z, 0JH605Z, 0JH606Z, 0JH60PZ, 0JH63PZ, 0JH80PZ, 0JH83PZ, 0JH634Z, 0JH804Z, 0JH834Z, 0JH635Z, 0JH805Z, 0JH835Z, 0JH636Z, 0JH806Z, 0JH836Z, 02H40NZ, 02H43NZ, 02H44NZ, 02H60NZ, 02H63NZ, 02H64NZ, 02H70NZ, 02H73NZ, 02H74NZ, 02HK0NZ, 02HK3NZ, 02HK4NZ, 02HL0NZ, 02HL3NZ, 02HL4NZ

Supplemental Table S2: Utilization of Therapeutic Cardiac Procedures After Multivariate Adjustment Among Hospitalized STEMI Patients, Stratified by COVID-19 Status

**Outcome**	**COVID-19 Positive**	**COVID-19 Negative**	***p*-value**
Systemic thrombolytic therapy	4.2%	1.1%	<0.001
	Adjusted odds ratio: 3.2 (95% CI: 2.2 - 4.6)		
Percutaneous coronary intervention	32.9%	70.1%	<0.001
	Adjusted odds ratio: 0.29 (95% CI: 0.25 - 0.34)		
Coronary artery bypass grafting	0.95%	4.1%	<0.001
	Adjusted odds ratio: 0.27 (95% CI: 0.14 - 0.51)		
Percutaneous left ventricular assist device	1.2%	2.9%	0.001
	Adjusted odds ratio: 0.38 (95% CI: 0.21 - 0.68)		
Intra-aortic balloon pump	4.2%	6.5%	0.01
	Adjusted odds ratio: 0.62 (95% CI: 0.43 - 0.90)		
